# Community pharmacists’ experiences in mental illness and addictions care: a qualitative study

**DOI:** 10.1186/s13011-016-0050-9

**Published:** 2016-01-28

**Authors:** Andrea L. Murphy, Heather Phelan, Scott Haslam, Ruth Martin-Misener, Stan P. Kutcher, David M. Gardner

**Affiliations:** College of Pharmacy, Dalhousie University, 5968 College St., PO Box 15000, Halifax, NS B3H 4R2 Canada; School of Nursing, Dalhousie University, 5869 University Ave., PO Box 15000, Halifax, NS B3H 4R2 Canada; Sun Life Financial Chair in Adolescent Mental Health, Dalhousie University/IWK Health Centre, 5850 University Ave., PO Box 9700, Halifax, NS B3K 6R8 Canada; Department of Psychiatry, Dalhousie University, QEII HSC, AJLB 7517, 5909 Veterans’ Memorial Lane, Halifax, NS B3H 2E2 Canada

**Keywords:** Pharmacist, Theoretical domains framework, Mental illness, Addiction, Qualitative

## Abstract

**Background:**

Community pharmacists are accessible health care professionals who encounter people with lived experience of mental illness and addictions in daily practice. Although some existing research supports that community pharmacists’ interventions result in improved patient mental health outcomes, gaps in knowledge regarding the pharmacists’ experiences with service provision to this population remain. Improving knowledge regarding the pharmacists’ experiences with mental illness and addictions service provision can facilitate a better understanding of their perspectives and be used to inform the development and implementation of interventions delivered by community pharmacists for people with lived experience of mental illness and addictions in communities.

**Methods:**

We conducted a qualitative study using a directed content analysis and the Theoretical Domains Framework as part of our underlying theory of behaviour change and our analytic framework for theme development. The Theoretical Domains Framework facilitates understanding of behaviours of health care professionals and implementation challenges and opportunities for interventions in health care. Thematic analysis co-occurred throughout the process of the directed content analysis. We recruited community pharmacists, with experience dispensing psychotropics, at a minimum, through multiple mechanisms (e.g., professional associations) in a convenience sampling approach. Potential participants were offered the option of focus groups or interviews.

**Results:**

Data were collected from one focus group and two interviews involving six pharmacists. Theoretical Domains Framework coding was primarily weighted in two domains: social/professional role and identity and environmental context and resources. We identified five main themes in the experiences of pharmacists in mental illness and addictions care: competing interests, demands, and time; relationships, rapport, and trust; stigma; collaboration and triage; and role expectations and clarity.

**Conclusions:**

Pharmacists are not practicing to their full scope of practice in mental illness and addictions care for several reasons including limitations within the work environment and lack of structures and processes in place to be fully engaged as health care professionals. More research and policy work are needed to examine better integration of pharmacists as members of the mental health care team in communities.

**Electronic supplementary material:**

The online version of this article (doi:10.1186/s13011-016-0050-9) contains supplementary material, which is available to authorized users.

## Background

Pharmacists’ roles in the community pharmacy setting in Canada and elsewhere in the world are expanding. Professional role revision in health care occurs in response to factors including, but not limited to, demands of the public and changing expectations, challenges with accessibility, advances in technologies and treatments, shortages of health care professionals, and increased health care costs [1]. In mental illness and addictions care, many of these factors co-occur alongside broader changes in the funding and delivery of health care such as shorter hospitalizations, fewer inpatient beds, and increasing demands on physicians and other primary care providers to care for people with serious mental illnesses and addictions [2–5]. Ensuring that all disciplines are practicing to their full potential and scope in mental illness and addictions care is one of many mechanisms to facilitate overcoming these, and various other challenges, such as enduring issues with timeliness, accessibility, appropriateness of care, and continuity of care [5–7].

Pharmacists can impact various outcomes (e.g., improve adherence rates, decrease prescribing of potentially inappropriate psychotropics) in mental illness and addictions care [8, 9]. However, attempts to capitalize on the potential benefits of pharmacists working within their full scope of practice in mental illness and addictions care presents both opportunities and challenges. A recent review [10] highlights opportunities for pharmacists in mental illness and addictions care regarding the kinds of services they can offer, how they offer them, and where they are delivered. For example, pharmacists are increasingly participating in multi-disciplinary teams, providing services outside of community pharmacy contexts in what would be considered non-traditional settings for a pharmacist (e.g., patient homes, primary care clinics), and engaging in activities beyond direct dispensing duties (e.g., depression screening) [10, 11]. Challenges to enhancing pharmacists’ roles include factors specific to the pharmacist, their practice environment, the patients, or other variables that may limit pharmacists’ abilities in providing quality care in keeping with standards of practice and the preferences of people with lived experiences of mental illness and addictions. Examples of these factors can include stigma, limitations within the context of community pharmacy practice (e.g., staffing, unpredictable workflow, privacy issues, lack of time), and issues with knowledge, skills, and competence in medication management for mental illness and addictions or other areas such as communication [12–18]. Limited or nonexistent remuneration for pharmacists’ services has also been reported as an impediment to mental illness and addiction service delivery in community pharmacies in other areas of the world [18].

Inherent in the pharmacist’s role are medication dispensing-related activities. Despite increasing prevalence of psychotropic prescriptions, medications with the potential for misuse, and addiction pharmacotherapies globally, and especially in Western cultures, research regarding the community pharmacists’ experiences in the care of people with lived experience of mental illness and addictions is limited. The majority of literature in mental illness and addictions care has focused on the patients’ experiences or outcomes (e.g., adherence, satisfaction) as a result of pharmacists’ interventions [8–10, 13, 16, 19–34]. Studies of pharmacists in mental illness and addictions care are primarily conducted using syntheses and survey methods and have focused on: characterizing pharmacists’ roles; assessing pharmacists’ attitudes towards patients and treatments; and obtaining perceptions around educational needs [9, 10, 16, 35–44]. Surveys can contribute valuable information and breadth regarding issues with pharmacy service provision, but available qualitative studies [18, 45] can allow for greater depth of understanding around the pharmacists’ experiences and behaviours in the context of their practice along with relevant challenges and opportunities. This knowledge is extremely useful in designing interventions aimed to enhance pharmacy-based care of people with lived experience of mental illness and addictions.

In our program of research, we have been working to develop programs and interventions informed by an understanding of how and under what circumstances pharmacists can effectively and efficiently engage in providing services to people with lived experience of mental illness and addictions in the context of the community pharmacy setting. A critical component to intervention design and development includes first understanding the behaviours of pharmacists in the context of their practice and therefore, our work is underpinned by various behaviour change theories and frameworks, including the Behaviour Change Wheel (BCW) and the Theoretical Domains Framework (TDF) [46–49]. This includes questioning who needs to do what differently, when, where, and how, and examining why the behaviours are enacted as they are in context [47]. We also need to consider what likely needs to change for the target or desired behaviours to occur [47]. Use of these theories supports developing interventions and programs with targeted behaviour change techniques that can overcome potential barriers to the successful implementation for these programs [47].

Based on our knowledge of the existing literature examining pharmacists’ roles in mental illness and addictions care, we determined that conducting a study using qualitative methods was appropriate to better inform the design and development of interventions or programs aimed at enhancing pharmacists’ roles in mental illness and addictions care. Underpinning the conceptualization, design, and analysis of the study with behaviour change theory allows for a more systematic process for developing our understanding of the behaviours in context and providing us with a better foundation in moving forward with future intervention design. We therefore conducted a qualitative study to gain and extend knowledge and understanding of the pharmacists’ experiences and behaviours in the practice context in providing services to people with lived experience of mental illness and addictions in the community pharmacy setting underpinned by behaviour change theory [46, 47] and the TDF [48].

## Methods

### Design

**Fig. 1:**
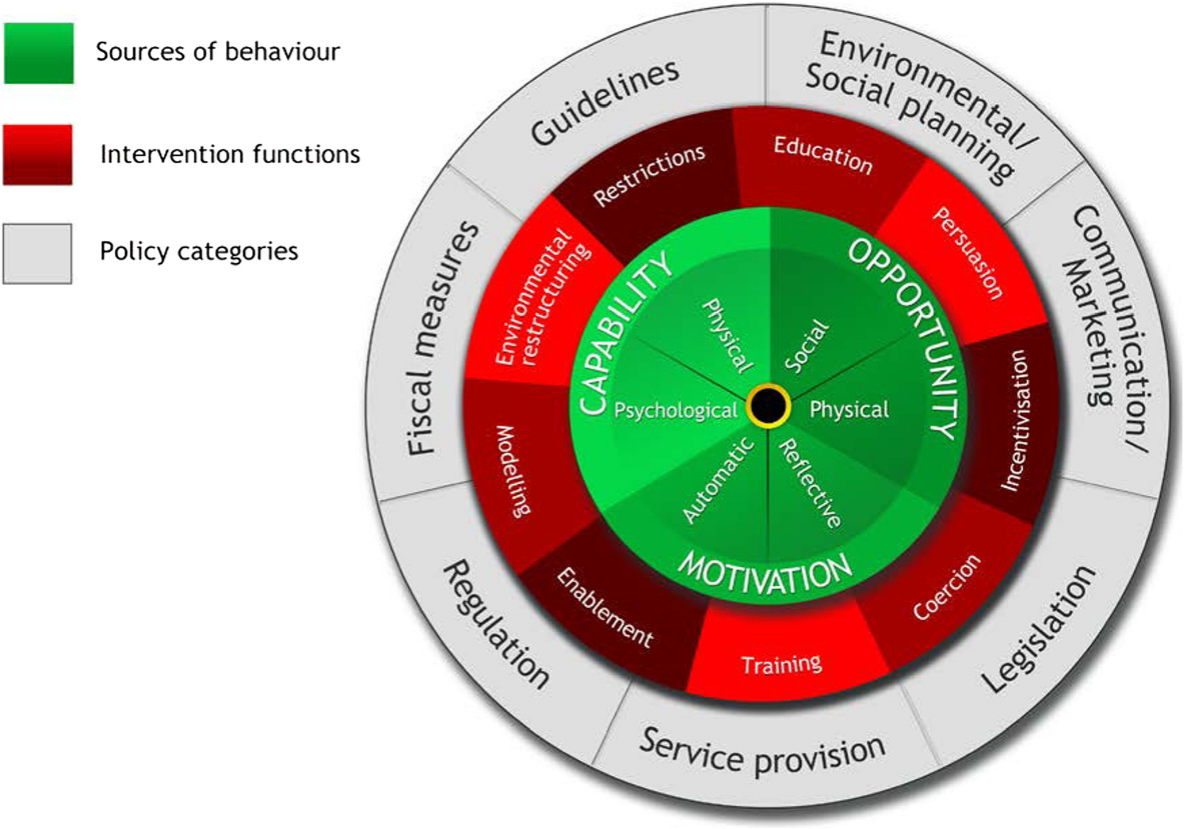
The Behaviour Change Wheel [47]. From Michie et al. [47], BioMed Central, Implementation Science

We conducted a qualitative study using directed content analysis [50, 51] guided by the TDF (see Table 1 for domains), which can be used to understand behaviours of health care professionals and implementation challenges and opportunities for interventions in health care [48]. The TDF was developed from 128 constructs from 33 psychological theories through an expert consensus process [52]. It was originally developed with 12 domains [52] and further modified to include 14 domains and 84 component constructs [48]. The philosophy behind the development of the TDF was to make psychological theories relevant to behaviour change and more accessible for disciplines, other than psychology, who are involved in the design, development, implementation, and evaluation of interventions in health care [52]. The TDF has been used to understand behaviours and implementation challenges in a variety of settings and content areas in health care (e.g., transfusion medicine, emergency care, hand hygiene, prescribing errors) [53] and more recently, but to a limited extent, by pharmacists [12, 54]. The TDF is a refined version of the framework for understanding behaviour known as the COM-B (capability, opportunity, motivation, and behaviour), which is at the centre of the Behaviour Change Wheel [47]. Information gained from research using the TDF can be used to help us understand behaviours and challenges and opportunities in practice, and also inform future intervention design by facilitating the use of the BCW (Fig. 1) [47]. The TDF domains have been mapped onto the COM-B (Table 1) [47, 48] at the centre of the BCW. To illustrate, environmental context and resources of the TDF are mapped to the physical opportunity in the COM-B and therefore, interventions or behaviours that are occurring because of specific issues within the environmental context reflect on limitations in the physical opportunity to engage in the behaviours. For example, a cardiovascular risk reduction program for people with lived experience of mental illness is implemented in the pharmacy but has little uptake by pharmacists because of lack of time and difficulty experienced by pharmacists in determining which patients would benefit most because of inefficient workflow and the diversity of patients at the pharmacy. Using the Behaviour Change Wheel, we can then determine potential intervention functions and policy categories that can be used in intervention designs to overcome issues that have been identified towards the desired behaviours [47]. Issues as described earlier within the environmental context with lack of time and uncertainty as to which patients would benefit are amenable to restriction, environmental restructuring, and enablement as intervention functions, based on the work of Michie et al. [47]. Therefore, as in the example of the cardiovascular risk reduction program with little uptake, environmental restructuring could include a computer report or on-screen prompts to alert pharmacists to people at highest risk for cardiovascular disease based on an algorithm with imputed risk factors (e.g., age, medications that have the potential for cardiometabolic side effects (e.g., some antipsychotics)). In this example, the pharmacists are taught to use prompts/cues, which is considered one of numerous potential behaviour change techniques [47, 55, 56].

**Table 1:** Theoretical Domains Framework [48] coding frequency of pharmacists’ experiences in providing services to people with lived experience of mental illness and addictions

Domain	Corresponding part of COM-B^a^ system from the Behaviour Change Wheel [47]	Coding frequency
Social/Professional Role and Identity	Motivation (reflective)	130
Environmental context and resources	Opportunity (physical)	67
Social influences	Opportunity (social)	38
Beliefs about capabilities	Motivation (reflective)	38
Skills	Capability (physical)	28
Knowledge	Capability (psychological)	26
Optimism	Motivation (reflective)	21
Emotion	Motivation (automatic)	17
Beliefs about consequences	Motivation (reflective)	12
Reinforcement	Motivation (automatic)	8
Goals	Motivation (reflective)	6
Intentions	Motivation (reflective)	2
Memory, attention, and decision processes	Capability (psychological)	2
Behavioural regulation	Capability (psychological)	-

^a^COM-B: C is capability, O is opportunity, M is motivation, and B is behaviour

Our work in this study is situated in the early stages of intervention design, in which we attempt to understand the pharmacists’ experiences and behaviours in context with the use of the TDF, which, as previously stated, is a refined version of the framework for understanding behaviour (i.e., the COM-B) in the Behaviour Change Wheel.

### Study participants

We used convenience sampling and recruited pharmacists using multiple mechanisms including advertisements through professional association newsletters, the Internet (e.g., classified advertisement sites such as Kijiji), and word-of-mouth. Pharmacists were eligible to participate if they were licensed to practice and had experience in providing pharmacy-based services, which included dispensation of psychotropics, at a minimum, to people with lived experience of mental illness and addictions. Participants were paid a one-time honorarium for participation.

### Data collection and analysis

A semi-structured interview guide (Additional file 1) was drafted by two research team members (ALM, DMG) after reviewing the literature and based on tacit knowledge of mental health care and community pharmacy practice. The draft was sent to other team members (RMM, SPK) for review and feedback. Based on feedback, the guide was refined and revised and subsequently pilot-tested with four senior pharmacy students, a medical student with a Masters degree and research background in mental illness and addictions care, and two community pharmacists. All were given background information regarding the purpose and objectives of the project. Questions were examined for understandability, relevance, clarity, and fitness for purpose. Comments and feedback were collected and revisions to the guide were made prior to finalization.

Recruitment occurred during June and July of 2012. Pharmacists were offered the option of interviews or focus groups for pragmatic reasons given our knowledge of the challenges with scheduling and staffing in pharmacies. We recognized that focus groups and interviews would give different kinds of information. For the purpose of our study and intended use of the findings for future intervention design, the integration of data would give complementary views of the phenomenon as well as the potential for greater depth and breadth [57]. All encounters with participants were digitally audio-recorded and subsequently transcribed. Transcriptions were read twice along with the audio for cleaning and anonymizing of personal information of pharmacists and patients. All data were collected during June through August of 2012. We aimed to recruit at least ten pharmacists to achieve saturation with TDF coding given the scope of our study and our intention to use information from the TDF coding and themes in program and intervention development [58, 59].

A directed content analysis approach was used with the TDF and its constructs serving as the coding scheme for the codebook. We adhered to the definitions of the 14 domains, and 84 constructs within these, as described by Cane et al. [48], and these served as our coding definitions. Four members of the team (ALM, HP, SH, DMG) coded the transcripts using directed content analysis. Prior to coding each transcript, we familiarized ourselves with the data through reading the transcripts several times to develop “a sense of the whole beyond the immediate initial impression” (p.143) [60]. We then read and coded one transcript initially and then met to discuss coding. We reviewed the TDF domains and provided exemplars from our coding to ensure we had clarity regarding meaning. We proceeded to code the remaining transcripts and met following the completion of each to discuss coding and main themes surrounding the TDF coding. Intercoder reliability based on percentage agreement was between 80 and 90 % for codes.

Two team members (ALM, DMG) were responsible for theme development. We followed modified procedures outlined for thematic analysis [61, 62]. The analysis stages, which co-occurred with our directed content analysis, included [61, 62]: familiarizing ourselves with the data; deductively coding and “flagging” meaningful data elements with each transcript analysis [60]; and searching for themes, reviewing themes, defining and naming themes through bringing coded data together and “interrogating” [60] relationships among TDF codes and themes. Theme development occurred independently at first and subsequently over the course of six, 1 to 2-hour meetings, themes were refined, debated, and finalized. All members of the team reviewed and critiqued the findings. Data were managed with QSR NVivo 10 [63].

### Ethics

Ethics approval was received from Capital District Health Authority Research Ethics Board (CDHA-RS/2013-042), which has reciprocal ethics in place with Dalhousie University Research Ethics.

## Results

We conducted one focus group (66 min) with four participants and two interviews (44 and 54 min) with practicing pharmacists (*n* = 2 males, *n* = 4 females;164 min of total data). Three staff pharmacists, one owner, and two managers participated, and all were from different pharmacies. Participants characterized their practice as urban (*n* = 4), suburban (*n* = 1), and rural (*n* = 1) based on general population estimates. The mean participant age was 35 years (range 29 to 43). The average hours worked per week was 41 (range 35 to 50) and years in practice ranged from 6 to 17 (mean 9.2). Monthly psychotropic prescription proportion estimates ranged from 30 to 80 %. Five pharmacists reported regularly dispensing methadone maintenance therapy. Four additional pharmacists were interested in participating initially but did not; one could not be reached to determine the reason for absence and three canceled just prior to the session due to conflicts.

### Theoretical domains framework

The most commonly occurring codes from the TDF were social/professional role and identity and environmental context and resources (Table 1).

### Themes

We identified five key themes from pharmacists’ experiences of caring for people with lived experience of mental illness and addictions including: 1) competing interests, demands, and time; 2) relationships, rapport, and trust; 3) stigma; 4) collaboration and triage; and 5) role expectations and clarity. Although individual themes are separate and important in their own right, there are linkages among themes in the experiences of pharmacists. For example, evidence supporting competing interests, demands and time and stigma were also linked with impacting the abilities of the pharmacists and patients in forming relationships, establishing rapport, and trust.

1. Competing interests, demands, and time
  Pharmacists in the community pharmacy setting were challenged to balance competing interests and demands on their time and many of these centred around business practices. Most of the supporting information from this theme came from codes within environmental context and resources, social professional role and identity, and to a lesser extent social norms. All pharmacists mentioned not having enough “time” for patients, which they acknowledged was necessary for caring for all patients but especially for those with lived experience of mental illness and addictions. The lack of time was a representation of many underlying issues such as a lack of resources with staffing, an inability to predict or control workflow, and inefficiencies in the workflow process. Issues with human resources and managing workflow directly affected their abilities to provide care and attention for those with mental illness and addictions. Regardless of the pharmacist’s role within the pharmacy (i.e., owner, manager, staff pharmacist), they were keenly aware of how competing interests and demands for their time and attention impacted their care of patients vis-à-vis the business aspects of pharmacy practice.
  Pharmacists discussed ideas, including different compensation models, to improve issues, albeit with skepticism and recognition of various tensions:
  “FGR1 (Focus Group Respondent 1): … it’s a weird profession we’re in. We’re in a health profession as well as retail. And so it’s hard to reconcile those two. … Yes, I think if you would have more compensation for the time. But I don’t really see how that would work very effectively the way that things are run now. FGR2: If you did have, though, fee-for-service, you could schedule in appointments for people. You know, so that you could have an extra pharmacist there in the morning. The pharmacist, because they’re getting paid extra, could go off [to be with the patient], take extra time. If people would be willing like to make an appointment to come in to talk about their medication. And I think people probably would, especially the people that come to the pharmacy all the time. FGR3: If they weren’t paying for it. FGR4: Oh, yes, they’re not paying for it, no. But if drug plans and social services and stuff recognized and paid for it. FGR3: I just don’t see them ever paying for it because that’s pretty much part of our job, is to like go through the medication. I don’t think they’re going to pay extra for spending that extra little time.”
  One pharmacist, who changed jobs to move into an ownership role, described her previous experience as a staff pharmacist with little control and power in managing competing demands. She was asked to use an abbreviated counselling approach (i.e., “Reader’s Digest” version) to provide more time for revenue generating activities such as filling prescriptions:
  “Interview Respondent (IR) 5: I wanted to be a pharmacy owner, just not to be told how I’m going to spend my time or how I’m going practice pharmacy. I’ll do it the way I want to do it. … when I worked in [town] … I was criticized for spending too much time with customers. And if I heard one more time about how I had to deliver the Reader’s Digest version. … I hated that. I would spend enough time. And sometimes people needed more time than others.”
  These abbreviated styles for patient counseling and education were reflected in the use of pharmacists’ language with “spew”, “spiel”, “bare bones”, “cut if off”, etc.:
  “FGR3: … you can get all your main points out [during counselling]. You take time to do that. You just don’t have time to sit there and chat with them and give … the rapport … encouragement and whatnot that they might need. … FGR2: Yes, it’s the bare minimum. … what the medication is for, the directions, side effects … And just really bare bones and if they have any questions. Then you’ve got someone else waiting for you. … you kind of have to spew out what you need to say. FGR3: … if it’s not busy, you can be like, “So how are you feeling?” … But most of the cases, it’s busy. … you just have to cut it [the discussion] off. FGR4: … It’s definitely dependent on how busy it is. … if you have three or four people at the counter, it’s hard to manage it. FGR3: And when I think back a couple of years ago when we did have [pharmacist] overlap … I would be more apt to do like follow-up calls, callbacks to see how they were making out. And now that just pretty much doesn’t exist. Which is unfortunate.”
2. Relationships, rapport, and trust
  All pharmacists acknowledged the importance of building relationships with people with lived experience of mental illness and addictions and consistently used words like “rapport” and “trust”. This was seen as essential in the pharmacist-patient relationship for accepting advice and getting “buy-in”:
  “FGR2: … if you can build up the rapport with the individual, and you get buy-in … then what you say has more weight with them. So maybe when you do talk to them about things, that, you know, you’ve built up this trust and then they kind of go with that more if you have the time to build up that relationship. But if you don’t have that then, you know, you’re just another face in a white coat.”
  This was echoed by another pharmacist discussing issues around addictions to medications including items available for purchase without a prescription (e.g., acetaminophen with codeine or Tylenol**®** No. 1) and building confidence in getting “buy-in” before refusing sales of these items:
  “IR5: I have one person that has alcoholism and I’d see her buying a fair amount of Tylenol 1 s. … I transferred some prescriptions for her … lithium and … I think I made some Antabuse**®** for her. And then of course that opened up the discussion … we talked a little bit about the Tylenol**®** with codeine use, and [I] asked her how she was using it. … that kind of prompted the discussion that … I certainly can’t keep her from buying it because she can go elsewhere, but was she okay with me not selling it to her anymore, because I didn’t feel comfortable selling it to her. I wasn’t helping her. And she really appreciated that. … But just trying to talk openly about it. … I don’t like the idea of just denying a sale just based on a suspicion if I haven’t got their buy-in. … if I haven’t discussed with them about … why I can’t sell it to them … I’m not doing them any favours by selling it to them. I’ve seen other alcohol addictions as well and usually people are pretty open about talking about that stuff with me. … I guess I’m more confident doing that [discussing acetaminophen and codeine use] because I had an incident when I worked in another town, and I had a young man come to me … he used to buy a lot of Tylenol**®** with codeine so we had the conversation about … what he was using it for. And it was chronic back pain. … I saw enough of him to know that he was using it pretty regularly. But then after maybe a year or 2 years, he came to me after being in rehab and telling me that he had a problem. He was asking me not to sell it to him anymore. … So that helped me to be more open in terms of asking people … I’m not always feeling like I’m intruding. I feel my intention is to help them, and it will come across that way.”
  The pharmacists recognized the importance of privacy and confidentiality in rapport and relationships and this was also tied in with stigma:
  “IR6: … people don’t want to stand in the middle of the aisle and talk [about] how their antipsychotic is working. But they wouldn’t mind saying, you know, how is your pain medication for your back that you threw out last week? So it’s a different kind of a sensitivity. … I tend to take those people, like I mentioned, over off to the side somewhere or to our private counseling area, and just have a chat with them. … They require a little bit more privacy for a conversation before they’re willing to open up.”
  The lack of privacy in the pharmacy environment significantly impaired some pharmacists’ abilities in their relationships with patients:
  “FGR2: It’s extremely difficult. … I was counseling a patient today on … a new antidepressant and a new antipsychotic. And I don’t even have like the barriers [privacy partitions]. … Meanwhile, there’s like three patients at the counter. … I have a sign posted – “Please respect patient’s privacy and stand back”. But nobody respects it … it makes the patient probably not as apt to either listen or want to hear.”
  Descriptions of relationships with people identified as having addictions were different than those without addictions. They were discussed in a manner best described as strained and tentative and pejorative terms were used more often. Stigma was also linked within these excerpts but the predominant theme was relationship, rapport, and trust. At times, there was mistrust discussed by pharmacists about their patients and their perceptions that patients were not trusting of the pharmacist. There was also a lack of clarity around whether patients actually had an addiction such as in many examples provided of patients misusing prescription medicines. One pharmacist in the focus group discussed “awkwardness” in the relationship with those experiencing addictions:
  “FGR3: … but when I first mentioned about the awkwardness, you don’t get that with many patients except for the addicts, and sometimes the mental health patients who are abusing their drugs and not using them properly. I don’t know if you’d classify them as addicts but if they’re using benzos or something like that for their anxiety then, you know, they might be using them more than appropriate. So you’d get that kind of awkward interaction that you wouldn’t get with people on a heart medication.”
3. Stigma
  Stigma and its impact on patient care and relationships was concerning to pharmacists. Pharmacists also spoke of their own views that may have been influenced by a stigmatizing culture and how this evolved through their education and training to becoming a practitioner and working with patients:
  “IR6: … you hear the word antipsychotic or you hear the word bipolar disorder and it kind of makes you envision the kind of person who’s going to require these medications. But they are, you know, for the most part, very “normal” individuals … when I did graduate, it was kind of taking the blinders off of what does mental illness actually look like and what does it not necessarily look like.”
  One pharmacist in the focus group assumed the patient’s perspective to consider what they would do in relationships with health care professionals based on a culture of stigma and if the public perceived a limited role for the pharmacist:
  “FGR2: … if I had like a mental health issue, I wouldn’t just open up to everybody about it because there’s a stigma about people with mental health issues. … “there’s something wrong with them…” or whatever the stigma is. So if I then don’t see my pharmacist as a health professional, if I see them as a pill counter, then I’m not going to open up to them. … when I go to a doctor’s office, my doctor is my health professional and that’s who I’m talking about my issues with. If I’m just picking up my pills then I’m not going to get into what I’m all about.”
  The issues of stigma were also discussed in terms of other supports for the patients including community and family. Participants practicing in “small” communities discussed having more challenges with stigma. The impact of stigma on patients in their homes was discussed and one pharmacist described their approach and solutions to mitigate this stigma, such as discussing information with family members to educate and inform them.
  “IR5: If they’re comfortable with their family being there then we try to have the discussion with everybody. Because again, there’s always the stigma. You know, you can talk with the patient all you want and give them a good understanding but if they have to go home to an environment where somebody might be saying, “… you’re crazy, you’re out of your mind, you don’t need those [medications],” or whatever.”
  Some of the language used by pharmacists during the focus group demonstrated that they relied on externalizing features and the kinds of medications people used to characterize patients who may require certain services for mental health and addictions problems:
  “FGR4: … it’s based on prescriptions that come in. … their appearance sometimes. For people dependent on opioids, their appearance is a little different from the regular clientele. They could have scabs or skinnier … not as well kept kind of thing. … FGR1: Yes, their mannerisms too.”
4. Collaboration and triage:
  Pharmacists discussed collaborating with several health care professionals such as physicians and social workers. Other individuals and representatives of community groups including police were also discussed. The nature of collaboration was often presented in the context of needs of the moment or in a reactive fashion. Successful collaborations were often the result of serendipity. The desire to have resources and knowledge of appropriate mechanisms for triage and community resources was evident.
  “FGR4: … things like refills, liaising with the doctor about medication issues. Then sometimes it’s just plugging them into other resources that are out there available. … if they’re [patients] coming in saying, “Well, I need to get this,” then you can refer them to the social worker or there’s the [organization], the [community group]. … A lot of times I find they run into issues with the [prescription] co-pays with social services. So there’s a lot of back and forth with the social workers …”
  One pharmacist described a serendipitous, snowballing approach to successful collaborations and triaging patients based on needs:
  “IR5: … I know I had a young girl who … could benefit from a psychologist. So I called and just tried to make some contacts for her … to get her in to have services. … through people that I met … people in mental health and psychologists, and then just going from there and making the phone calls. … [I] make the phone call and then have them steer me in the right direction. … I’ve always had good luck … that they know how to point me in the right direction. … I think I was very fortunate to have met the kinds of people that I did in my profession. … initially starting off years and years ago, that mental health nurse and then the psychologists [I met] along the way. … if we’re talking about building teams … it would be great to have these [education] programs with other individuals. … nursing and psychologists and with doctors, everybody in the same room. … And mainly teaching us where to access help. … like who to call.”
  Because of pharmacists’ roles in dispensation of opioids and many other medications with the potential for misuse due to physical or psychological dependence, the pharmacists described collaboration with police but for some, this relationship was tense:
  “FGR3: I had a police officer, he had to get a search warrant because he had suspicions of one of our patients selling his [opioid]. But he was pretty harsh on me. He kept asking me and asking me. And I was like … “… no, you really need to go get your search warrant because I’m not answering any more questions.” … they can be a little bit tough if they suspect something of your patient. So that wasn’t a very positive experience with the police officer.”
5. Role expectations and clarity:
  Pharmacists discussed participating in many activities that are in their current scope of practice but with some grey areas and blurred lines as to the boundaries of pharmaceutical care and support extending outside of these bounds. Lack of clarity in role and boundaries was a phenomenon that created tension among some pharmacists. These situations were exemplified through stories of pharmacists being a listener for patients, either over the phone or in person, with and without direct relevance to medication-related duties. This was aptly demonstrated by a pharmacist’s interaction with a grieving patient:
  “IR5: … she had a lot that she wanted to talk through. And I was criticized [by other pharmacists] because I’m not a counsellor, I don’t have to have those kind of conversations with her, I should have broken it off a lot sooner. But if she wants to talk about her [relative], I’m going to let her talk about her [relative]. And my intention was never to be a counsellor for her. My intention was just to be a listener for her. … I was talking with her about her antidepressant but what she wanted to do was to talk about her [relative], I’d let her talk about her [relative]. Not for that long a period of time, and I wouldn’t be offering advice. …”
  In addiction-related care, pharmacists in the focus group discussed situations at length, in which they were an intermediary between patients and doctors related to the dispensation of controlled drugs and substances. They did not feel consistently supported in their role by other health care professionals or their patients as demonstrated in many examples. They also discussed at length the tools and techniques they used when “saying no” to patients seeking their prescription medication refills prior to their due date. This commonly included citing regulatory standards, reimbursement issues, or physician unavailability as reasons to postpone access to a controlled prescription medication. They also acknowledged variability in how pharmacists handled these situations with some pharmacists taking a more flexible approach and allowing early prescription refills. Communication issues with physicians were also noted in describing situations in which the physician was aware and had permitted more medication use than what was originally intended with the prescription but the pharmacist was not made aware.
  “FGR2: … she [patient] was like 14 days early on a 90-day supply [of clonazepam]. And she had put it on our computer-automated system. … the computer-automated system doesn’t know whether it’s a benzo or a narcotic. And she said, “Well, it told me it would be ready today.” And I was like, “Well, unfortunately… It is 14 days early.” … “The best I can do is call your doctor on Monday because this is a controlled drug so I can’t dispense it early.” … I did explain everything but she took off and she was really mad. … But that’s … a typical situation. Some people will handle it better than others. And perhaps having another pharmacist that may have dispensed it 14 days early that I work with might have created a bit of anger towards me. … It ruins your day because you don’t want anyone walking away from you like huffing and puffing and being angry. … FGR3: … It makes you a bit nervous because what if you then go and call the doctor and they’re like, “Oh, give it to them. That’s fine if they’re using a little bit extra.” Then it really makes you look like the bad person. FGR4: I disagree because I think that we’re still doing our job. … FGR2: But if they’ve been so mad at you and maybe they’re telling you … the doctor said they could take it more often sometimes, but you can’t take their word. And then the doctor does say, “Oh, yeah, I told her she could take it more often sometimes.” Then you’re just this person who’s made like a bad moment for everyone. FGR1: … I guess I’d be hiding behind the rules and regulations and say, “You know, I’d like to do it but I’ve got to do it this way or I’d lose my license.” FGR3: … This is my license on the line. FGR1: And they’d [patients] tend to get that. FGR2: Sometimes drug plans will save me too because drug plans won’t pay for stuff early. And the patient won’t be willing to pay for it. So that’s an extra … FGR1: … safeguard, yes.”
  The pharmacists further addressed the physicians’ roles in the pharmacist-physician and patient-physician relationship in various situations such as when patients were requesting medications early:
  “FGR3: Hopefully to support us. But they’re not all like that. Some of them just give in to the patients time and time again. FGR4: Even like the addiction specialists … I’m often on the phone with them and they’re … it must be a really tough thing to be a physician as well because maybe a patient that has, you know, an addiction to a narcotic says, “Well, if I don’t get my narcotics, I’m going to go off and do this.”… I guess to give in is the lesser of two evils. … often doctors are too easy to give in to patients that are chronically abusing and chronically getting their medications filled early with a different excuse each time. Or they spilled their methadone … I think the physicians are giving in too easy. Which sort of takes away from our role as well because we’re trying to control them. We’re trying to get them off this medication. FGR3: Yes. When you have those types of doctors, sometimes you don’t go that route because you know the doctor is going to say, “whatever”. So with those types, you kind of put notes in their file – “Don’t give early”… FGR2: But the patient will contact the doctor anyway.”
  Pharmacists then attempted to name this part of their role:
  “FGR4: Ethics. FGR1: Gatekeeping. [laughs] FGR2: Medication police, or trying to be. FGR3: Protecting patients. I mean I’ve had some patients come back when they’ve got maybe a little bit better and thank me for being on top of not giving them stuff, and that kind of thing. I mean it doesn’t cure them and they usually fall back into it but some patients are appreciative. So I guess you’re just trying to help them and protect them.”
  Pharmacists were often passive in their role, as if their role was peripheral to caring for people with lived experience of mental illness and addictions. Many of these quotes were grouped under social/professional role and identity and also social influences. This was most obviously demonstrated when pharmacists discussed patients’ diagnoses. Pharmacists were relying on medications and combinations of medications to determine, or as one pharmacist said, “guesstimate”, what diagnoses patients had versus inquiring directly about the indication:
  “FGR4: … Nobody has ever said to me I have depression or I have schizophrenia. I’ve never heard anybody say that to me. FGR3: You can ask them a couple of questions about what symptoms they’re experiencing or what the doctor has told them, and sometimes that will reveal a little bit of information. But for the most part, I feel like they just keep quiet and they want to listen and get out of there. Then they might call later with questions. It depends on the person. If they’re dropping off an antipsychotic [prescription], I don’t think that most of them are asking a thousand questions and willing to give a lot of information. FGR2: You just kind of guesstimate based on the therapy. … if they’re just getting maybe one antidepressant, that they maybe have minor depression or maybe anxiety … If they’re big into antipsychotics … then you would guesstimate that they have something more severe, more troubling. But you don’t very often get their actual diagnosis, I suppose.”

## Discussion

Our study is one of a few qualitative explorations of pharmacists’ experiences in mental illness and addictions care [12, 17, 18, 23, 45] and adds further understanding to the pharmacists’ experiences and behaviours in the context of practice. The findings point to significant challenges in practice. It is also uniquely underpinned by behaviour change theory and the application of the TDF as an analytic framework, which proved useful and effective for coding and theme development for the pharmacists’ experiences with people with mental illness and addictions. These findings can inform the choice of various intervention functions and policy categories used in the design and development of interventions for community pharmacists in mental illness and addictions care. Based on our work, consideration of several TDF domains in new program/intervention design and development, especially social/professional role and identity, environmental context and resources, beliefs in capabilities, and social influences, is required to support community pharmacy-based care of people with lived experience of mental illness and addictions.

Environmental context and resource issues were particularly influential in directing pharmacists in the care of their patients in our findings. This was especially evident in the theme competing interests, demands, and time. Issues with time, manifesting from workflow inefficiencies and inadequate staffing, and lack of privacy limited pharmacists’ ability to care for people with lived experience of mental illness and addictions beyond basic medication-related services. This finding is similar to what pharmacy students observed of their work environments with “business tension” directing many actions of pharmacist supervisors and preceptors [12]. Although several of the pharmacists in our study did not perceive providing any additional services other than medication counseling, compliance packaging, and opioid substitution treatment services, they did in fact provide examples of other valuable actions for patients including health care system navigation, triage support, and referrals to community groups and health care professionals. Many of the services were offered through both face-to-face and telephone interactions and outside the context of a medication-related issue. Although various methods have been used to quantify pharmacists’ activities [64] and how pharmacists perceive their workloads, [65, 66] we are not aware of any research that examines the non-medication related interventions by pharmacists such as health system navigation and making referrals for mental illness and addictions care. It would be valuable to examine these encounters between patients and pharmacists to determine the range and extent of activities that pharmacists are doing in non-medication consultations and what impact these activities have on patient outcomes, the pharmacy practice environment, including impacts on quality and safety, and the broader mental health system.

Issues related to pharmacists’ roles and identity were prevalent in our study and this was especially evident in discussions of people with addictions and within the theme of collaboration and triage. The nature of the relationships among participant pharmacists, prescribers, and patients was not entirely supportive or positive as pharmacists acted as the intermediary between patients and doctors, or as they labeled it, “medication police” and “gatekeeper”. The participants expressed the desire for better relationships and the need for more collaboration, communication, and consistency with other health care professionals and patients. Communication challenges between physicians and pharmacists have been found to be prevalent in the care of people with lived experience of addictions [41, 67]. Participants described various examples of communication issues and other complex problems with people with lived experience of addictions to over the counter products (e.g., acetaminophen with codeine), alcohol, benzodiazepines, and opioids. The participant pharmacists had differing perspectives on their role, but it was primarily limited to technical and legal aspects of dispensation. Similar to a survey of pharmacists in Indiana, US [38], the care provided by pharmacists for people with addictions was time intensive. Our findings provide additional insights into the extent and range of issues including the layered complexity in negotiating with patients and physicians and especially in time intensive activities around issues with medication supply (e.g., early medication refills with benzodiazepines and opioids, spilled doses of methadone). Similar issues have been reported by a sample of pharmacists providing opioid substitution treatment in Australia in which argumentative behaviours regarding supply such as takeaway doses, which are important for convenience and maintaining normality for many patients [68], were one of the more common problematic aspects in care [42].

Social influences in the context of the pharmacy environment are also important to note and especially when considering attitudes and behaviours of all pharmacy staff with respect to stigma. Group norms and conformity, including use of pejorative terminology, could contribute to the continued stigmatization of vulnerable groups of people. Although there has been progress made in lessening stigma in many areas of health care, there are still examples from the pharmacy context, which negatively impact the care of patients [33, 35, 45, 69–80].

The beliefs about capabilities domain, inclusive of constructs such as self-efficacy and professional confidence, was also prominently coded and within the themes around role expectations and clarity and relationships, rapport, and trust. Looking to future intervention development, capitalizing on aspects of intervention design that work to enhance reflective motivation hold promise for encouraging desired behaviours. For example, recent work using interactive educational workshops improved pharmacists’ self-efficacy for documentation of care, which will inherently be required for improved community pharmacy-based mental illness and addictions care [81]. Other research has also demonstrated that adopters of innovations in pharmacy service delivery are more likely to be those with higher perceived self-efficacy [82].

Participant pharmacists also discussed wanting more interdisciplinary education and training on the services available for patients, how to help patients access these services, and general therapeutic-based information on addictions and mental illnesses. This is an important area to further explore given pharmacists receive little formal training in addictions and that more than half of pharmacists may never refer patients to drug treatment programs [36] despite the fact that most pharmacists perceive prescription drug abuse, including opioids intended for pain, to be a problem in their practice setting [43]. Inadequacies in pharmacists’ competencies, attitudes, and behaviours have been cited as barriers to optimal care for people with lived experience of addictions [38, 45] and these domains may be improved by education and training. For example, additional education and training increased the likelihood that pharmacists provided patients with information on addiction treatments and care [44]. Based on our findings and the scope of the issues, there appears to be significant opportunity to improve addictions care by pharmacists in community pharmacy settings, but improving knowledge, attitudes, and behaviours through education and training is only a component of this complex area. Substantial improvements will be required in structures and processes in the community pharmacy setting and the broader health system, including those related to communication, collaboration, and following best-evidence based care in addiction practice among health care professionals, in order to better support community pharmacists in their role.

Finally, the care of patients by our pharmacists appeared to be restricted through passive and peripheral involvement with aspects of care, including having limited to no knowledge of the patients’ diagnoses. Current practice standards recommend that pharmacists keep information regarding medical conditions in patient profiles to ensure appropriateness of therapy and facilitate recommendations. Pharmacists use information on diagnoses not only to assess appropriateness but also to tailor education and advice about the use of medications and expectations around benefits and adverse effects that are often linked with diagnoses. Implementation of electronic health records that are used health system-wide may facilitate the pharmacists’ access to information such as diagnoses, up-to-date dispensing records, and laboratory values, but the utility of these records may be variable and dependent on a number of factors (e.g., privacy and confidentiality, accuracy of the information, pharmacist role, the practice environment, collaboration with other health care professionals) [83, 84]. More work is needed regarding better integration of community pharmacists from the periphery into mental health care delivery in community settings.

### Limitations

Given the nature of this research, there may be more than one interpretation of the data, including different approaches to coding and thematic development. Members of our team include pharmacists with views shaped by disciplinary socialization and intimate knowledge of the milieu of practice. We attempted to foster reflexivity by having multiple team members, including those with different backgrounds, and by writing our thoughts via journaling throughout the study with particular attention to our biases.

Using a directed content analysis approach with the TDF, as in this study, forces the categorization of some attributes and constructs that are closely related and necessary for pharmacists’ interactions with patients, into different domains. For example, behaviours related to knowledge and skills are categorized separately but when combined, as would be the case in training programs to improve pharmacists’ capabilities in mental illness and addictions care, they were coded 54 times. The separation of these may result in different behaviour change techniques, intervention functions, and policy categories used within intervention designs. Although both knowledge and skills fall within capability when mapped to the COM-B, physical capability is mapped only to skills. There is the possibility that skills may therefore not be addressed adequately in a new intervention that primarily uses education, as this intervention function is linked to improving knowledge (psychological capability).

The number of participants was small although we obtained rich data, saturated our TDF coding, and determined that more data would not necessarily provide helpful information for our goal of future intervention development. However, we may have erroneously concluded that we reached saturation. We recognize the concept of saturation is debated and can be “highly problematic” [60], depending on the research question, and we cannot presume that we have all necessary data to understand a clinical phenomenon in its entirety. In our assessing the reliability of our coding we also used percentage agreement, which does not account for random agreement among coders.

Using a technique such as maximum variation sampling may have given us more diverse participant representation. We also included data from both focus group and interviews in our analysis, which may produce different information such that interviews can often give depth on a topic and focus groups may provide breadth and are also inherently subject to “group think”. Given the small number of participants, anonymizing pharmacy names and type (i.e., franchise, independent, etc.) to protect confidentiality was necessary and therefore we did not comment on how the pharmacy type may have influenced behaviours, which has previously been shown to impact pharmacists’ practices [85].

## Conclusions

Five main themes characterize the pharmacists’ experiences of caring for people with lived experience of mental illness and addictions including: competing interests, demands, and time; relationships, rapport, and trust; stigma; collaboration and triage; and role expectations and clarity. Pharmacists are not practicing to their full scope of practice in mental health and addictions care for several reasons including limitations within the context of the work environment and lack of structures and processes in place to be fully engaged as health care professionals. More research and policy work are needed to examine better integration of pharmacists as members of the mental health care team in communities.

## Additional file

Additional file 1:
**Interview guide for pharmacists.** (DOC 33 kb)

## Abbreviation

TDF: theoretical domains framework
