# Supplementary material for: Community pharmacists’ experiences in mental illness and addictions care: a qualitative study
Source: Subst Abuse Treat Prev Policy. 2016 Jan 28;11:6. doi: 10.1186/s13011-016-0050-9 (PMC4730654; doi:10.1186/s13011-016-0050-9)
Supplement: Additional file 1: — Interview guide for pharmacists. (DOC 33 kb) [file 13011_2016_50_MOESM1_ESM.doc]

# Additional file 1 INTERVIEW GUIDE FOR PHARMACISTS

**Question 1.**

**Tell me about your experiences with patients/clients that have mental illness in the pharmacy setting. Tell**

**me about your experiences with those with addictions pharmacy setting.**

***Probing Questions:***

- - - Describe some of the patients you serve that would be considered to have mental illness and/or addictions.
    - Discuss how you determine what patients require services related to their mental health?

**Question 2. Describe the services your community pharmacy provides for patients, or caregivers of patients, with mental illness and/or addictions? Are these services the same for all mental illnesses and/or addictions.**

***Probing Questions:***

- - - Can you give an example(s)? What does it involve?
    - Are these services that you initiate or are the initiated at the company level?
    - Tell me about any differences that might exist in the services provided for a person with schizophrenia or opioid addiction compared to a person with anxiety or depression.

**Question 3. Describe the services your community pharmacy provides for patients, or caregivers of patients, with physical illness?**

***Probing Questions:***

- - - Can you give an example(s)? What does it involve?
    - Are these services that you initiate or are the initiated at the company level?
    - Discuss how you offer services for physical complaints to patients with mental illness.

**Question 4. Discuss any differences in the quality or quantity of services provided for patients, or caregivers of patients, with mental illness and/or addictions as compared to physical illness?**

***If a difference between mental and physical health services is identified, PROBE with:***

- - - Why do you think there difference exists between services for mental health and services for physical health?
    - Describe any variations in the differences among mental illnesses and/or addictions.

**Question 5. Describe for us what your ideal services would look like for your community pharmacy to offer to patients, or caregivers of patients, with mental illness and/or addictions.**

***Probing Questions:***

- - - Describe your vision of pharmacy services for patients with mental illness and/or addictions.
    - What are the challenges you foresee in providing these services?
    - What opportunities or supports do you see in providing these services?

**Question 6. Describe the health care team members you work with in providing your services to patients and caregivers of patients with mental illness and/or addictions.**

***Probing Questions:***

- - - Is the health care team composition different for people with mental health problems compared to people with physical health problems?
    - Discuss your impressions about whether team dynamics and functioning are similar or different when discussing your patients with mental health problems as compared to patients with physical health problems.

**Question 7. Describe your role in your relationship with the patient and/or caregiver.**

***Probing Questions:***

- - - What do you feel are your responsibilities in providing pharmacy services?
    - How would you describe your relationship with patients and caregivers with lived experience with mental illness?

**Question 8. Discuss how your education, training, and continuing education have prepared you to work with people with mental illness and/or addictions.**

***Probing Questions:***

- - - Describe your needs in this area.
    - How do you meet your needs in this area?
